# NLS-tagging: an alternative strategy to tag nuclear proteins

**DOI:** 10.1093/nar/gku869

**Published:** 2014-09-26

**Authors:** Guillaume Giraud, Ralph Stadhouders, Andrea Conidi, Dick H.W. Dekkers, Danny Huylebroeck, Jeroen A.A. Demmers, Eric Soler, Frank G. Grosveld

**Affiliations:** 1 Department of Cell Biology, Erasmus Medical Center, Faculty building, PO Box 2040, 3000 CA Rotterdam, The Netherlands; 2 Proteomics Center, Erasmus University Medical Center, Faculty building, PO Box 2040, 3000 CA Rotterdam, The Netherlands; 3 Laboratory of Molecular Biology (Celgen), Department of Development and Regeneration, KU Leuven, Herestraat 49, B-3000 Leuven, Belgium; 4 Laboratory of Hematopoiesis and Leukemic Stem Cells (LSHL), CEA/INSERM U967, Fontenay-aux-Roses, France; 5 Center for Biomedical Genetics and Medical Epigenetics Consortium, Erasmus Medical Center, Faculty building, PO Box 2040, 3000 CA Rotterdam, The Netherlands; 6 Center for Biomedical Genetics, Erasmus Medical Center, Faculty building, PO Box 2040, 3000 CA Rotterdam, The Netherlands

## Abstract

The characterization of transcription factor complexes and their binding sites in the genome by affinity purification has yielded tremendous new insights into how genes are regulated. The affinity purification requires either the use of antibodies raised against the factor of interest itself or by high-affinity binding of a C- or N-terminally added tag sequence to the factor. Unfortunately, fusing extra amino acids to the termini of a factor can interfere with its biological function or the tag may be inaccessible inside the protein. Here, we describe an effective solution to that problem by integrating the ‘tag’ close to the nuclear localization sequence domain of the factor. We demonstrate the effectiveness of this approach with the transcription factors Fli-1 and Irf2bp2, which cannot be tagged at their extremities without loss of function. This resulted in the identification of novel proteins partners and a new hypothesis on the contribution of Fli-1 to hematopoiesis.

## INTRODUCTION

Transcription factors (TFs) regulate gene expression through their recruitment to gene regulatory sequences (1). They often function as protein complexes cooperating with other TFs or cofactors to regulate many biological processes, such as cellular proliferation and differentiation. For example, protein complexes containing the Ldb1 TF have been shown to control erythroid differentiation by regulating the expression of key erythroid-specific genes (2).

Much of our current knowledge of the molecular mechanisms TF use to regulate gene expression comes from the identification of their genomic binding sites by chromatin immunoprecipitation (ChIP) experiments and the identification of their protein partners by pull-down assays usually followed by mass spectrometry (MS) analysis to determine the identity of the co-precipitated factors. These approaches rely on the efficient and specific purification of the proteins and DNA bound by the factor of interest using antibodies. The availability of high-affinity antibodies against particular TFs is, therefore, critical for experimental success. These experiments are usually single-step purifications and/or are performed on low number of cells. The antibodies should therefore be efficient and very specific to obtain a high signal-to-noise ratio to allow the identification of true DNA/protein or protein/protein interactions. However, suitable antibodies are often not available at all or perform suboptimally. A popular alternative to antibodies is therefore the generation of a fusion between a small epitope ‘tag’ sequence and the protein of interest because purification strategies for these are readily available. These short peptide sequences, which are either recognized by high-affinity antibodies or by streptavidin (biotag), have been widely used alone or in combination to characterize TF complexes and genome-wide binding sites (3–5). The peptide tag is fused to either the N-terminal or to the C-terminal end of the protein, however, the addition of extra amino acids to one or both termini can disrupt protein function and/or its stability, as exemplified by the Myef2 protein (6). Because most proteins are modular in structure, an alternative strategy to circumvent problems with terminal tagging would be to integrate the tag sequence next to a domain within the protein (7,8). Several constraints need to be respected for this approach. Most importantly, the tag should not be integrated in a functional domain of the protein, which is often not well defined. Moreover, the tag should be positioned in a region of the protein that is expected to be highly exposed to the cellular milieu in order to promote recognition by antibodies or by the BirA enzyme. Again, such information is usually not available. We therefore thought of using a domain that is almost ubiquitously present and accessible in TFs, namely, the nuclear localization signal (NLS).TFs contain a NLS recognized by the importin α/importin β heterodimers that transport the protein from the cytoplasm through the nuclear pore into the nucleus (9). This domain will be exposed in all cells where the TF is active, although it can be regulated by post-translational modifications (e.g. phosphorylation) or by NLS masking. A well-studied example of the latter is the control of NF-κB nuclear import that is regulated by its interaction with IκB, which masks the NF-κB NLS to prevent its nuclear import (10). Together with structural studies of the FUS NLS (11), the data indicate that the NLS forms an exposed site on the protein that can be recognized by the importin complex.

Here, we address the possibility to make use of the exposed NLS for tagging purposes by integrating a tag sequence close to the NLS as an alternative for the classical C-/N-terminal approach and used two ‘difficult’ proteins, Fli-1 and Irf2bp2, to test this strategy. A 3×Flag-biotin peptide was integrated close to the NLS of these TFs, whose C-/N-terminal tagging disrupt their function (data not shown). Their expression in an erythroid progenitor cell line (which also expresses these protein endogenously) showed that their function is unaffected. We then used the NLS-tagged Fli-1 protein to identify its protein partners by MS analysis in erythroid cells for the first time and found novel protein partners belonging to the key erythroid Ldb1 TF complex.

## MATERIALS AND METHODS

### Plasmid constructs

Expression vectors for 3×Flag-Bio(NLS)-Fli-1 and 3×Flag-Bio(NLS)-Irf2bp2 were obtained by stepwise insertion of Fli-1 and Irf2bp2 cDNA parts into a modified pBud plasmid containing the 3×Flag sequence. First, the N-terminal coding part up to the NLS sequence was inserted followed by the insertion of the C-terminal coding part containing the bio-tag. The different coding parts have been obtained by polymerase chain reaction (PCR) amplification from a MEL cells cDNA library using the primers listed in Supplementary Table S2. Constructs were verified by Sanger sequencing before transfection in MEL/BirA cells (12). Clones were selected using neomycin at 1 mg/ml. Only clones expressing the same level as the endogenous protein were used for analysis.

### Cell culture

MEL cell lines were maintained in Dulbecco's modified Eagle's medium supplemented with 10% fetal calf serum and penicillin/streptomycin. Cells were induced by culturing them in presence of 2% of di-methyl sulphoxide (DMSO) for 4 days.

### Nuclear extracts

Cells were washed twice with 1× phosphate buffered saline (PBS) and resuspended in 8 ml of cold buffer A (10 mM HEPES-KOH pH 7.9, 1.5 mM MgCl2, 10 mM KCl, 0.5 mM DTT, 1× Complete ethylenediaminetetraacetic acid (EDTA)-free protease inhibitor mix (Roche)). After 10 min of incubation in ice, lysates were vortexed and spun down 10 s at 13 200 revolutions per minute (rpm) at 4°C. Pellets were resuspended in 1 volume of buffer C (20 mM HEPES-KOH pH 7.9, 25% glycerol, 420 mM KCl, 1.5 mM MgCl2, 0.2 mM EDTA, 0.5 mM DTT, 1× Complete EDTA-free protease inhibitor mix (Roche)) and incubated 20 min in ice. Lysates were vortexed and pelleted 2 min at 13 200 rpm at 4°C. The supernatant was used for further experiments.

### Co-immunoprecipitation

Co-immunoprecipitation experiments were performed starting from 1 mg (2 mg for MS) of nuclear extracts prepared as mentioned above and diluted to 150 mM KCl with Heng0 buffer (20 mM HEPES-KOH pH 7.9, 20% glycerol, 0.25 mM EDTA pH 8.0, 0.05% NP-40, 1× Complete EDTA-free protease inhibitor mix (Roche)). Note that 10 μl of magnetic beads (Dynabeads M-280, Life Science) (blocked during 1 h in presence of 0.2 μg/μl Chicken Egg Albumin) coated with streptavidin for 1 mg of proteins are added to the nuclear extracts together with 100 U benzonase (Roche). After 3 h of incubation at room temperature, the beads were washed five times with Heng150 (20 mM HEPES-KOH pH 7.9, 20% glycerol, 0.25 mM EDTA pH 8.0, 0.2% NP-40, 150 mM KCl, 1× Complete EDTA-free protease inhibitor mix (Roche)) for 5 min at room temperature and directly resuspended in 2× Laemmli buffer (120 mM Tris-HCl, pH 6.8, 4% sodium dodecyl sulphate (SDS), 20% (w/v) glycerol, 0.01% bromophenol blue, 400 mM DTT). Finally, the beads were boiled 5 min and discarded. Co-immunoprecipitation experiments using the anti-Flag antibody (M2, Sigma-Aldrich) were performed as mentioned above except that the nuclear extracts were incubated with 10 μg of antibody and 10 μl of magnetic beads coated with protein G for 4 h at room temperature.

### Western blot

Nuclear extracts or immunoprecipitated proteins were boiled in Laemmli buffer and loaded onto NuPAGE precast 4–12% gradient Bis-Tris acrylamide gels (Invitrogen). Proteins were transferred to nitrocellulose membranes and probed for the protein of interest using the antibodies mentioned below. Fluorescently labeled secondary antibodies (Licor) were used for visualization and membranes were scanned on an Odyssey Imaging System.

The following primary antibodies were used : Rabbit anti-Fli-1 (Abcam, ab-15289), Rat anti-Gata-1 (Santa Cruz Biotechnology, N6), Rabbit anti-Irf2bp2 (Absea), Rabbit anti-Klf1 (5-V) (kindly provided by Dr. Sjaak Philipsen), Mouse anti-Vcp (Abcam).

### Quantitative reverse transcriptase-PCR (RT-PCR)

Total RNA was extracted from the different cell lines using TRI Reagent (Sigma). First-strand cDNA was synthesized using the SuperScript II First Strand Synthesis System (Invitrogen) and oligo-dT primers (Invitrogen). Real-time PCR was performed using Platinium Taq Polymerase and SYBR green (Invitrogen) cDNA on a Bio-Rad CFX96 PCR System. Ribonuclease/angiogenin 1 (*Rnh1*) was amplified in parallel for normalization purposes. Primer sequences are listed in Supplementary Table S3.

### ChIP followed by real-time PCR

Note that 1 × 10^7^ cells were cross-linked 50 min at room temperature using DSG (Di(N-succinimidyl) glutarate) 50 mM (Proteochem), washed twice with 1× PBS followed by an additional cross-linking step of 10 min using 1% formaldehyde (Merck). The cross-linking reaction was stopped by adding 0.125 M Glycine. Cells were washed twice with 1× cold PBS and resuspended in sonication buffer (Tris-HCl 10 mM pH8.0, EDTA 1 mM, EGTA 0.5 mM). After 10 min of incubation in ice, chromatin was sonicated for 33 cycles (15 s ON, 30 s OFF Amp 9) using the Soniprep 150 (Beun de Ronde) to get fragments of 500–800 bp. The sonicated chromatin from 10^6^ cells was then diluted 20 times in ChIP dilution buffer (0.01% SDS, 1.1% Triton X-100, 1.2 mM EDTA, 16.7 mM Tris-HCl pH 8.0, 167 mM NaCl) and incubated overnight in presence of 10 μl of magnetic beads (Dynabeads M-280, Life Science) (blocked for 1 h with 1.5% fish skin gelatin and 200 ng sonicated salmon sperm DNA). The beads were then washed once with Low Salt Buffer (0.1% SDS, 1% Triton X-100, 2 mM EDTA, 20 mM Tris-HCl pH 8.0, 150 mM NaCl), once with High Salt Buffer (0.1% SDS, 1% Triton X-100, 2 mM EDTA, 20 mM Tris-HCl pH 8.0, 500 mM NaCl), once with LiCl Buffer (0.25 M LiCl, 1% NP-40, 1% deoxycholixacid sodium salt, 1 mM EDTA, 10 mM Tris-HCl pH 8.0) and twice with TE buffer (10 mM Tris-HCl pH 8.0, 1 mM EDTA). The beads were then decrosslinked in presence of Elution Buffer (1% SDS, 0.1 M NaHCO_3_) during 4 h at 65°C and treated for 1 h with 20 μg proteinase K. Finally, DNA was purified.

ChIP using the anti-Fli-1 (Santa Cruz Biotechnology, sc-356) was performed using the same protocol as described above except for the following differences. The diluted sonicated chromatin was incubated for 30 min with agarose beads coated with protein A and G (Santa Cruz Biotechnology). After spinning 1 min at 1000 rpm at 4°C, the chromatin of 10^6^ cells was incubated overnight at 4°C with either 10 μg of anti-Fli-1 antibody or the same amount of Rabbit immunoglobulin G (IgG; Santa Cruz Biotechnology). Agarose beads coated with protein A and G were then added and the samples were incubated for 1 h at 4°C on a rotating wheel.

### Immunofluorescence

Cells were sediment for 15 min on a poly-L-lysine coated glass slide for 15 min at room temperature and fixed for 15 min with 2% PBS/paraformaldehyde at room temperature. Cells were then quickly washed three times with PBS/0.1% Triton-X100 and twice with the same buffer for 10 min. After an extra quick wash with PBS/0.5% bovine serum albumin (BSA)/0.15% Glycine, cells were incubated for 2 h at room temperature with streptavidin conjugated with Alexa Fluor 488 diluted 250 times with PBS/0.5% BSA/0.15% Glycine. Cells were quickly washed three times with PBS/0.1% Triton-X100, washed for 10 min with same buffer and finally quickly washed with 1× PBS. A total of 10 μl prolong GOLD were added to the cells and a coverslip was mounted. After 24 h overnight at room temperature, slides were visualized in the confocal microscope Lyca SP5.

### Mass spectrometry

The beads containing bound TF complexes (see Co-immunoprecipitation section) were washed twice with 50 mM NH_4_HCO_3_ and incubated overnight at 37°C with shaking with 0.1 μg of trypsin/50 μl beads. The digests are analyzed by nanoflow liquid chromatography-tandem MS on a LTQ-Orbitrap (Thermo) mass spectrometer coupled to an 1100 series LC pump and autosampler (Agilent) operating in positive mode and equipped with a nanospray source. Peptide mixtures were trapped on a ReproSil C18 reversed phase column (Dr. Maisch GmbH; column dimensions 1.5 cm _ 100 μm, packed in-house) with a flow rate of 8 l/min. Peptide separation was performed using a ReproSil C18 reversed phase column (Dr. Maisch GmbH; column dimensions 15 cm _ 50 μm, packed in-house) using a linear gradient from 0% to 80% B (A = 0.1 M formic acid; B = 80% (v/v) acetonitrile, 0.1 M formic acid) over 70 min with a constant flow rate of 200 nl/min using a splitter. The column eluent was directly sprayed into the electron spray ionization (ESI) source of the mass spectrometer (13). Mass spectra were acquired in continuum mode; while fragmentation of the peptides was performed in data-dependent mode. Peak lists were automatically created from raw data files using the Mascot Distiller software (version 2.1; MatrixScience). The Mascot search algorithm (version 2.2, MatrixScience) was used for searching against the NCBInr database (latest NCBInr release; taxonomy: *Mus musculus*). The peptide tolerance was typically set to 10 ppm and the fragment ion tolerance to 0.8 Da. A maximum number of two missed cleavages by trypsin were allowed and carbamidomethylated cysteine and oxidized methionine were set as fixed and variable modifications, respectively. The Mascot score cut-off value for a positive protein hit was set to 80 (5).

## RESULTS

### Generation of MEL cells expressing NLS-tagged Fli-1 and Irf2bp2 proteins

**Figure 1.**
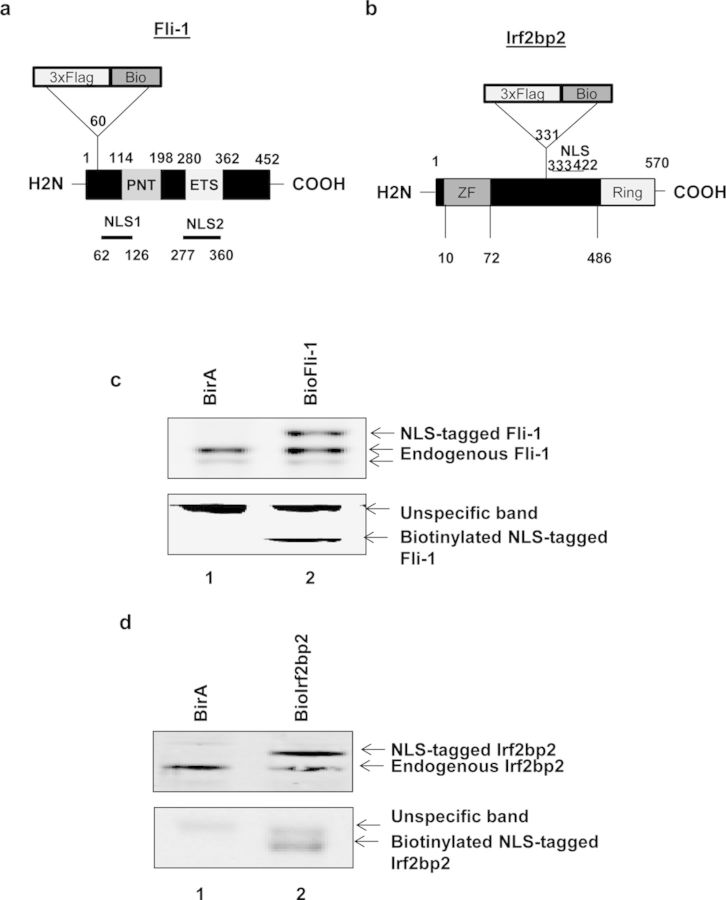
Expression of NLS-tagged Fli-1 and Irf2bp2 proteins in MEL cells. **(a)** Schematic depicting Fli-1 protein domain organization and the 3×Flag-Bio tag integration site. The Fli-1 pointed (PNT) domain is involved in the interaction with other proteins, the ETS domain is the DNA binding domain. Fli-1 has two NLSs localized between aminoacids 62 and 126 (NLS1) and between aminoacids 277 and 360 (NLS2). The 3×Flag and BirA target sequence (Bio) were integrated in between amino acids 60 and 61, just before the NLS1. **(b)** Schematic depicting Irf2bp2 protein domain organization and the 3×Flag-Bio tag integration site. Irf2bp2 contains one zinc finger (ZF) and one RING domain. The NLS of this factor is localized between amino acids 333 and 422. The 3×Flag and Bio sequences were integrated in between amino acids 331 and 332. **(c** and **d)** Total proteins were extracted from either MEL/BirA, MEL/BioFli-1 (c) or MEL/BioIrf2bp2 (d) cells and subjected to Western blot analysis. Membranes were probed using an antibody against the endogenous proteins (top) or using streptavidin (bottom).

Fli-1 is expressed as a 51 and 48 kDa protein isoform that belongs to ETS family of TFs. Like all ETS TFs, it contains an ETS DNA binding domain of around 85 amino acids involved in the recognition of a GGAA core consensus sequence (14). Fli-1 contains two NLSs: the first NLS is located between amino acids 62 and 126 while the second is localized between amino acids 277 and 360 that correspond to the ETS domain (Figure 1a) (15). We therefore decided to integrate the popular triple FLAG (3×FLAG) and a biotin tag (biotag) between amino acids 60 and 61 in a region that does not contain any known domain critical for Fli-1 function (Figure 1a).

Irf2bp2 is a 60 kDa nuclear protein found to function as a co-repressor of the IRF2 TF (16). The Irf2bp2 NLS has been mapped between amino acids 333 and 422. Teng *et al.* showed that nuclear localization of Irf2bp2 is dependent on the phosphorylation of the serine 360 residue (17). We therefore decided to integrate the 3×FLAG and a biotag between amino acids 331 and 332, which do not belong to any important domain of the Irf2bp2 protein (Figure 1b).

Fli-1 and the Irf2bp2 cDNA containing the tag in the aforementioned positions were cloned in a modified pBud vector that allows cDNA expression driven by a EF1α housekeeping gene promoter. These constructs were transfected into MEL cells that express the BirA biotin ligase (MEL/BirA) at level sufficient to biotinylate TFs carrying a biotag (2,12). Stable clones expressing the NLS-tagged proteins were selected based on their expression levels. Total protein extracts were analyzed by western blot using either an anti-Fli-1 antibody (Figure 1c, top panel), an anti-Irf2bp2 antibody (Figure 1d, top panel) or directly using streptavidin (Figure 1c and d, bottom panel).

Several clones expressing 3×Flag-Bio(NLS)-Fli-1 or 3×Flag-Bio(NLS)-Irf2bp2 were obtained (data not shown). Figure 1c and d show that MEL/BioFli-1 and MEL/BioIrf2bp2 cells express the NLS-tagged proteins at similar levels as the endogenous protein. Moreover, streptavidin western blot revealed the presence of specific bands at the expected size of 3×Flag-Bio(NLS)-Fli-1 or 3×Flag-Bio(NLS)-Irf2bp2 in these stable cell lines (Figure 1c and d, bottom panel).

These data show that the NLS-tagged factors can be expressed at the same level as the endogenous proteins. Moreover, tag integration near the NLS allows recognition and biotinylation of the proteins by BirA biotin ligase.

### NLS-tagged Fli-1 and Irf2bp2 are properly localized in the nucleus

The integration of the tag sequences close to the NLS of Fli-1 or Irf2bp2 proteins may disrupt their nuclear localization. Hence, we first performed immunofluorescence assays using streptavidin conjugated with Alexa-Fluor 488 and Dapi in MEL/BirA, MEL/BioFli-1 and MEL/BioIrf2bp2 cells.

**Figure 2.**
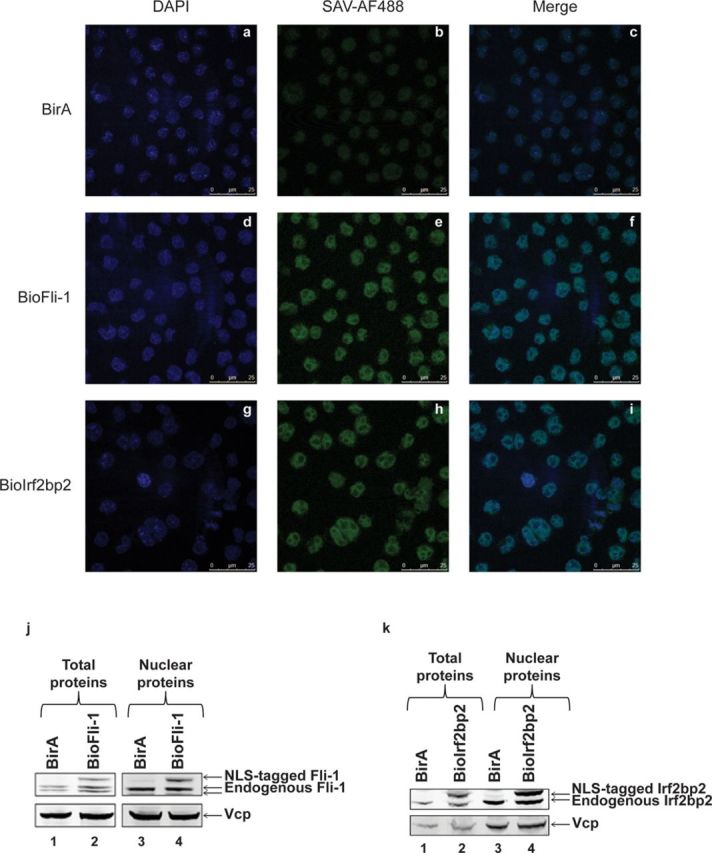
Proper nuclear localization of the NLS-tagged proteins in MEL cells. **(a–i)** Immunofluorescence experiments in MEL/BirA (a, b, c), MEL/BioFli-1 (d, e, f) and MEL/BioIrf2bp2 (g, h, i) cells using either DAPI (a, d, g) or streptavidin conjugated with Alexa fluor 488 (b, e, h). The figure c, f and i show the merged picture. **(j** and **k)** Total (lanes 1 and 2) and nuclear (lanes 3 and 4) proteins were extracted from MEL/BirA (lanes 1 and 3), MEL/BioFli-1 (j, lanes 2 and 4) and MEL/BioIrf2bp2 (k, lanes 2 and 4) and subjected to Western blot analysis. Membranes were probed using an antibody against the endogenous protein (top panel) or against Vcp (bottom panel, loading control).

While no signal was observed in the nucleus using Streptavidin in MEL/BirA control cells (Figure 2b and c), Figure 2e and f show a clear signal, which merged with DAPI in MEL/BioFli-1 cells indicating that the NLS-tagged Fli-1 protein is, as expected, localized in the nucleus. Similarly, the NLS-tagged Irf2bp2 protein is also localized in the nucleus (Figure 2h and i).

To determine if the NLS-tagged proteins have a similar nuclear localization as the endogenous protein, we compare the ratio between the exogenous and the endogenous protein in total protein and nuclear protein extracts of MEL/BioFli-1 and MEL/BioIrf2bp2 cells. Western blot analyses shown in Figure 2j (compare lanes 2 and 4) and k (compare lanes 2 and 4) indicate that the ratio between the NLS-tagged proteins and the endogenous protein is similar in total protein extracts compared to nuclear extracts showing that the NLS-tagged proteins have a similar nuclear localization as the endogenous protein.

Altogether, these results indicate that integration of a 3×Flag and a biotag sequence close to the NLS of Fli-1 and Irf2bp2 proteins does not disrupt their nuclear localization.

### NLS-tagged Fli-1 and Irf2bp2 interact with known protein partners of the endogenous protein

Even though NLS-tagged proteins are properly localized in the nucleus, integration of a short peptide sequence within the Fli-1 and Irf2bp2 proteins could possibly affect their interaction with other proteins. To test this possibility, we investigated whether 3×Flag-Bio(NLS)-Fli-1 or 3×Flag-Bio(NLS)-Irf2bp2 are able to interact with known protein partners of the endogenous proteins.

**Figure 3.**
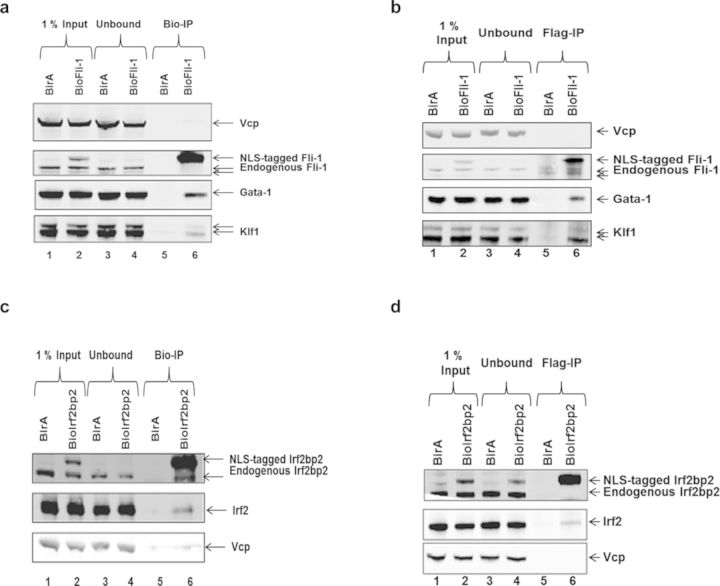
NLS-tagged Fli-1 and Irf2bp2 interact with endogenous interacting proteins. **(a)** Streptavidin-IP on nuclear extracts from MEL-BirA (lanes 1 and 3) or MEL/BioFli-1 cells (lanes 2 and 4) followed by Western blot analysis of Vcp (top panel, loading control), the endogenous and the NLS-tagged Fli-1 proteins (middle top panel), Gata-1 (middle bottom panel) and Klf1 (bottom panel). The picture is representative of two independent experiments. **(b)** Flag-IP of nuclear extracts from MEL/BirA (lanes 1 and 3) or MEL/BioFli-1 cells (lanes 2 and 4) followed by Western blot analysis of Vcp (top panel, loading control), the endogenous and the NLS-tagged Fli-1 proteins (middle top panel), Gata-1 (middle bottom panel) and Klf1 (bottom panel). The picture is representative of two independent experiments. **(c)** Streptavidin-IP of nuclear extracts from MEL/BirA (lines 1 and 3) or MEL/BioIrf2bp2 cells (line 2 and 4) followed by Western blot analysis of the endogenous and the NLS-tagged Irf2bp2 proteins (top panel), Irf2 (middle panel) and Vcp (bottom panel, loading control). The picture is representative of two independent experiments. **(d)** Flag-IP of nuclear extracts from MEL/BirA (lines 1 and 3) or MEL/BioIrf2bp2 cells (lines 2 and 4) followed by Western blot analysis of the endogenous and the NLS-tagged Irf2bp2 proteins (top panel), Irf2 (middle panel) and Vcp (bottom panel). The picture is representative of two independent experiments.

We first tested whether the NLS-tagged Fli-1 protein interacts with Gata-1 and Klf1, two known Fli-1 protein partners identified in MEL cells and megakaryocytes (18,19). Streptavidin-IPs were performed in MEL/BirA control cells, which only express the BirA biotin ligase and in MEL/BioFli-1, which express the NLS-tagged Fli-1 protein. The absence of the NLS-tagged Fli-1 protein in the unbound fraction shows that more than 90% of the NLS-tagged Fli-1 protein is biotinylated (Figure 3a, compare lanes 2 and 4). Besides, while the streptavidin-IP in MEL/BirA cells does not pull-down Gata-1 or Klf1 (lane 3), the same experiment in MEL/BioFli-1 cells precipitates Gata-1 (line 4, middle panel) and Klf1 (lane 4, bottom panel) together with 3×Flag-Bio(NLS)-Fli-1 (line 4, top panel). The same experiments using the antibody against the 3×Flag tag in MEL/BirA and MEL/BioFli-1 cells show that Gata-1 and Klf1 are also specifically precipitated in MEL/BioFli-1 cells (Figure 3b). These results show that, like the endogenous Fli-1 protein, NLS-tagged Fli-1 interacts with Gata-1 and Klf1 despite the presence of the internal tag sequences.

As mentioned before, Irf2bp2 was first discovered as a protein partner and corepressor of the IRF2 TF (16). To check if the NLS-tagged Irf2bp2 also interacts with Irf2, similar experiments as above were performed in MEL/BirA and MEL/BioIrf2bp2 cells. The results shown in Figure 3c and d indicate that NLS-tagged Irf2bp2 indeed interacts with Irf2 in MEL cells.

Taken together, these results show that the integration of short sequences close to the Fli-1 and Irf2bp2 NLS does not disrupt their interaction with other proteins.

### NLS-tagged Fli-1 and Irf2bp2 are recruited to known binding regions of the endogenous Fli-1 and Irf2bp2 proteins

TFs modulate gene expression through their binding to gene regulatory regions. We therefore also investigated whether the integration of a tag close to the NLS does not disrupt the DNA binding pattern of NLS-tagged Fli-1 and Irf2bp2 by performing ChIP experiments using streptavidin-conjugated beads (BioChIP).

**Figure 4.**
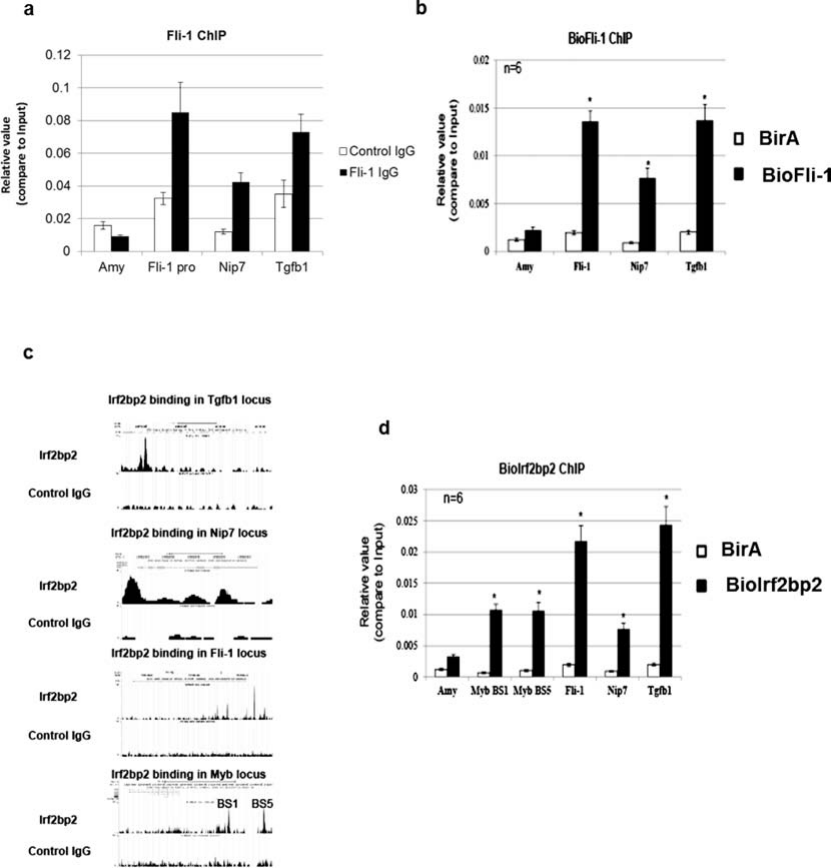
NLS-tagged Fli-1 and Irf2bp2 are recruited to the endogenous protein target regions. **(a)** ChIP experiments with the anti-Fli-1 antibody (black bars) or the Control IgG (white bars) from MEL cells followed by qPCR using primers amplifying *β-amylase* (control region), *Fli-1* promoter, *Nip7* promoter and a region within the *Tgfb1* locus. Data represents the average of three independent experiments; error bars denote standard deviation. **(b)** Streptavidin-ChIP from MEL/BirA (white bars) and MEL/BioFli-1 (black bars) cells followed by qPCR using primers amplifying *β-amylase* (control region), *Fli-1* promoter, *Nip7* promoter and a region within the *Tgfb1* locus. Data represents the average of six independent experiments; error bars denote standard deviation, *P < 0.05, Student's *t*-test between MEL/BirA and MEL/BioFli-1 cells. **(c)** Genome-wide Irf2bp2 binding sites in MEL cells were identified by ChIP-Seq experiments. The different Irf2bp2 genomic binding regions can be visualized by the UCSC genome browser. For example, Irf2bp2 binds a region within the *tgfb1* locus, the *nip7* gene promoter, the *fli-1* gene promoter and two known enhancers of *c-myb* gene (BS1: Myb −36 kb; BS5: Myb −68 kb). **(d)** Streptavidin-ChIP from MEL/BirA (white bars) and MEL/BioIrf2bp2 (black bars) cells followed by qPCR using primers amplifying *β-amylase* (control region), *Myb −36 kb (BS1)*, *Myb −68 kb* (BS5), *Fli-1* promoter, *Nip7* promoter and a region within the *Tgfb1* locus. Data represents the average of the signal for six independent experiments; error bars denote standard deviation, *P < 0.05, Student's *t*-test between MEL/BirA and MEL/BioIrf2bp2.

Some target regions of Fli-1 have been reported in MEL cells using the anti-Fli-1 antibody. Fli-1 was shown to be recruited to its own promoter (20,21), to the *nip7* gene promoter, which encodes a protein involved in ribosome biogenesis (22). We confirmed the recruitment of Fli-1 to these specific regions and also to the *tgfb1* locus by performing ChIP experiments using the anti-Fli-1 antibody in MEL cells. Although the efficiency of the ChIP is variable between the biological triplicates, we observed a higher enrichment with the anti-Fli-1 antibody compared to the Rabbit IgG for all these three regions (Figure 4a). We then tested whether 3×Flag-Bio(NLS)-Fli-1 could also be recruited to these loci in MEL/BioFli-1 cells. BioChIP experiments performed on these cells and MEL/BirA control cells showed a specific enrichment in these three loci only in MEL/BioFli-1 cells, with no enrichment observed in the *β-amylase* control locus (Figure 4b). Besides, the efficiency of the BioChIP is more reproducible than the ChIP with the anti-Fli-1 antibody. These results indicate that NLS-tagged Fli-1 is also recruited to the *fli-1* and *nip7* promoters as well as the *tgfb1* locus in MEL cells.

We recently determined the genome-wide Irf2bp2 binding sites by ChIP-Seq in MEL cells using an antibody raised against the endogenous protein (Soler, in preparation). Interestingly, Irf2bp2 is also recruited to the Fli-1 occupied loci mentioned above and also binds to two known enhancers of *Myb* (23) (Figure 4c). Similar to Fli-1, we performed BioChIP experiments in MEL/BioIrf2bp2 and MEL/BirA control cells to verify whether the NLS-tagged Irf2bp2 protein is also recruited to these specific loci. Figure 4d indeed shows the recruitment of NLS-tagged Irf2bp2 to five known binding regions of the endogenous Irf2bp2 protein.

Altogether, these results show that the presence of a short tag sequence close to the Fli-1 and Irf2bp2 NLS does not affect their recruitment to gene regulatory regions.

### NLS-tagged Fli-1 inhibits MEL cells differentiation

Fli-1 overexpression and depletion experiments performed in mouse and human cells demonstrated that Fli-1 is a repressor of erythroid differentiation (21,22,24). Specifically in MEL cells, the overexpression of Fli-1 represses erythroid differentiation of these cells (21). To further validate the functionality of the NLS-tagged Fli-1 protein, we examined the potential of 3×Flag-Bio(NLS)-Fli-1 to repress the DMSO-induced erythroid differentiation of MEL cells.

**Figure 5.**
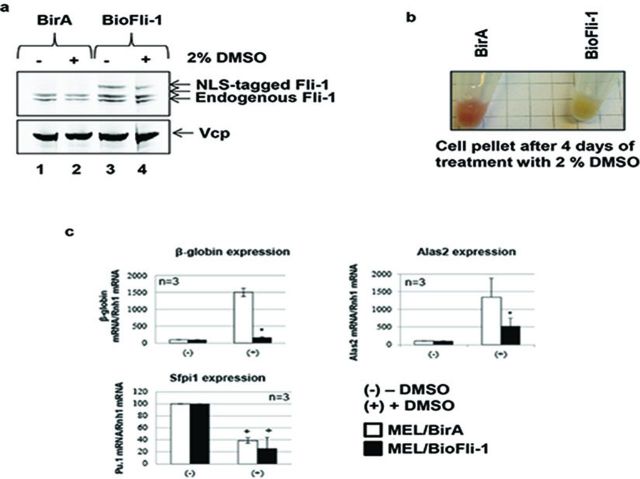
NLS-tagged Fli-1 inhibits erythroid differentiation of MEL cells. **(a)** Total proteins extracted from MEL/BirA (lanes 1 and 2) and MEL/BioFli-1 (lanes 3 and 4) cells cultured for 4 days in presence (lanes 2 and 4) or in absence (lanes 1 and 3) of 2% DMSO were subjected to western blot analysis. Membranes were probed using an antibody against the endogenous protein (top panel) or the Vcp protein (bottom panel, loading control). The figure is representative of three independent experiments. **(b)** Pellet of MEL/BirA (left tube) and MEL/BioFli-1 (right tube) cells after 4 days of DMSO treatment. **(c)** RT-qPCR experiments on MEL/BirA (white bars) and MEL/BioFli-1 (black bars) cells measuring the expression of *β-globin* (top left panel), *alas2* (top right panel) and *sfpi1* (bottom panel). Data represents the average of three independent experiments; error bars denote standard deviation. *P < 0.05, Student's *t*-test between MEL/BirA and MEL/BioFli-1 cells. +*P* < 0.05, Student's *t*-test between untreated and DMSO treated cells.

As observed in Figure 5a, the 4-day DMSO treatment does not affect the expression of the NLS-tagged Fli-1. Control cells obtained a red color, a well-known sign of hemoglobin synthesis and erythroid differentiation (Figure 5b, left tube). In contrast, MEL/BioFli-1 cells remain white after the same treatment indicating that a functional 3×Flag-Bio(NLS)-Fli-1 is able to inhibit erythroid differentiation.

To confirm these observations, we measured the induction of *β-globin* and *Alas2* expression, two established markers for terminal erythroid differentiation, by RT-qPCR experiments. In contrast to MEL BirA control cells, MEL/BioFli-1 cells show either an impaired (*Alas2*) or no (*β-globin*) induction of erythroid gene expression upon treatment while both cell type show a decrease of *Sfpi-1* gene expression, an early effect of DMSO in MEL cells (25) (Figure 5c).

Altogether, these results demonstrate that NLS-tagged Fli-1, similar to the endogenous Fli-1 protein, represses the erythroid differentiation of MEL cells despite the integration of a short tag close to its NLS.

### Fli-1 interacts with several proteins belonging to the Ldb1 complex

The previous experiments described above show that the integration of a short epitope tag sequence next to the NLS of Fli-1 and Irf2bp2 does not affect their function. To take advantage of the epitope tag for protein purification, we used the MEL/BioFli-1 cells to purify Fli-1 protein partners in MEL cells by performing immunoprecipitation experiments using streptavidin-conjugated beads followed by MS in both MEL/BirA control cells and MEL/BioFli-1 cells.

**Figure 6.**
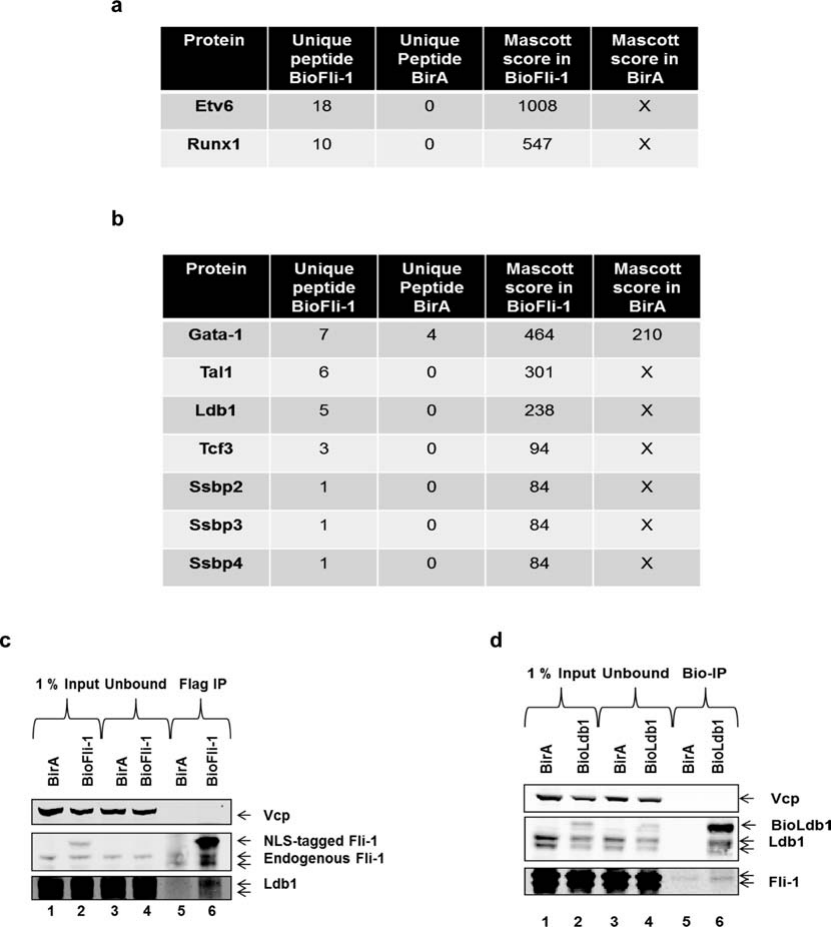
Fli-1 interacts with several members of the Ldb1 complex in MEL cells. **(a)** Streptavidin-IP from MEL/BirA (BirA) and MEL/BioFli-1 (BioFli-1) nuclear extracts followed by MS was used to identify new Fli-1 protein partners in MEL cells. Table shows the number of peptides detected in both MEL cells and their corresponding Mascott scores for two known Fli-1 interacting proteins : ETV6 (26) and Runx1 (27). **(b)** Table depicting several Fli-1 interacting proteins detected by MS belonging to the Ldb1 complex. **(c)** Flag-IP from MEL/BirA (BirA) (lanes 1, 3 and 5) and MEL/BioFli-1 (lanes 2, 4 and 6) nuclear extracts followed by Western blot analysis of Vcp (top panel, loading control), the endogenous and the NLS-tagged Fli-1 proteins (middle panel) and the Ldb1 protein (bottom panel). The picture is representative of two independent experiments. **(d)** Streptavidin-IP from MEL/BirA (BirA) (lanes 1, 3 and 5) and MEL/BioLdb1 (BioLdb1) (lanes 2, 4 and 6) nuclear extracts followed by Western blot analysis of Vcp (top panel, loading control), the endogenous and tagged Ldb1 proteins (middle panel) and the Fli-1 protein (bottom panel). The picture is representative of two independent experiments.

The full list of the identified proteins interacting with Fli-1 is shown in Supplementary Table S1. According to the MS results, Fli-1 interacts with 99 proteins. Among these proteins, some have been already identified by other groups in different cellular model system. For example, we found among the 99 interacting proteins, the ETS TF ETV6 (also called TEL) whose interaction with Fli-1 has been identified in human K562 cells (26). Moreover, we also found Fli-1 to interact with the Run×1 TF, an interaction found to be important for megakaryopoiesis (27) (Figure 6a). These results further validate the functionality of the NLS-tagged Fli-1 as a valuable tool to study TFs functions.

Strikingly, among the other proteins we found interacting with Fli-1, several members of the Ldb1 TF complex were detected (Figure 6b). Among these proteins, we found the core complex members Gata-1 (whose interaction was confirmed by streptavidin-IP experiments (Figure 3a), Tal1/Scl, Ldb1 itself, Tcf3/E2A and several Ssbp proteins). All Ldb1 complex members were found interacting specifically in 3×Flag-Bio(NLS)-Fli-1 cells (although Gata-1 was also found in the MEL/BirA purification, the Mascott score was less than 2-fold lower as compared to 3×Flag-Bio(NLS)-Fli-1 cells). We next performed new Flag-IP experiments in MEL/BirA and MEL/BioFli-1 cells to confirm the interaction between Fli-1 and Ldb1. As shown in Figure 6c, Ldb1 is pulled-down together with NLS-tagged Fli-1 while no signal corresponding to the Ldb1 protein was detected in MEL/BirA control cells validating our MS data. We also performed co-immunoprecipitation experiments using MEL cells expressing a bio-tagged Ldb1 (12) to check whether BioLdb1 interacts with Fli-1. As shown in Figure 6d, Fli-1 is precipitated more with bio-Ldb1 than in MEL/BirA control cells while similar amount of proteins were used in both cells.

In summary, these data indicate that the NLS-tagged Fli-1 protein is a functional protein that can be efficiently purified, allowing us to identify for the first time the critical erythroid Ldb1 TF complex as a Fli-1 protein interacting partner.

## DISCUSSION

Here, we describe a focused strategy of epitope tagging by integrating the sequence close to the NLS (‘NLS-tagging’) of two very different TFs that could not be effectively tagged at their N- or C-terminal end (data not shown). We have shown that the NLS-tagged proteins still interact with known protein partners, that they are still recruited to regulatory sequences bound by the endogenous proteins and that the NLS-tagged Fli-1, like the endogenous protein, is still able to repress the erythroid differentiation of MEL cells. Therefore, we propose NLS-tagging as an effective and fast alternative strategy to the common N- or C-terminal tag integration methods for the characterization of protein complexes formed by TFs and the identification of their DNA binding regions.

The NLS-tagging strategy allowed us to show that Fli-1 interacts with several members of the Ldb1 complex. This complex is mainly known for its positive regulation of erythropoiesis by being recruited to regulatory sequences of erythroid-specific genes (2). This TF complex is also formed in megakaryocytes where it regulates the expression of megakaryocytic genes and the megakaryocyte differentiation (28). These data highlight similar and opposite contributions of Fli-1 and the Ldb1 complex during megakaryopoiesis and erythropoiesis, respectively, resulting in a new hypothesis about the Fli-1 contribution during hematopoiesis. Altogether, these data show that our NLS-tagging strategy can greatly facilitate the elucidation of TF function during developmental processes, exemplified here by new insights in the contribution of the Fli-1 protein to hematopoiesis.

Epitope tag exposure is critical to efficient recognition of the tag by antibodies (or streptavidin in the case of the bio-tag). We chose close proximity to the NLS sequence as a suitable strategy here as the NLS sequence is an often highly exposed domain of the protein. Although we tested this on only two proteins, we expect this to work with many if not all TFs. As a consequence, however, the strategy we propose can only be readily used for TFs or other nuclear proteins with a known NLS, although the NLS can often be recognized from the amino acid sequence. There are several such sequences, the classical sequence PKKKRKV (29) for which the consensus K-K/R-X-K/R was proposed or the bipartite-type sequence KR[PAATKKAGQA]KKKK (30). Other sequences, such as the PY-NLS motif (proline-tyrosine pairing), have also been identified as a *bona fide* NLS binding importin β2 (31). In addition, the NLS of TF are usually present either in the DNA-binding domain or its vicinity making this region a likely target to integrate the tag (32). Other sequences involved in protein localization could also be chosen for internal tagging of nuclear proteins with an unknown NLS sequence or cytoplasmic proteins. For example, the NES (nuclear export signal) has to be recognized by proteins, such as CRM1, to export proteins from the nucleus to cytoplasm and as a consequence, this sequence should be exposed (33). Moreover, proteins have often modular structure. As we could see for Fli-1 and Irf2bp2 (Figure 1), they are composed of different functional domains important for their biological functions. Any place where domains are linked is potentially a tag sequence integration site. However, some will not work properly as they may be inside the protein. Finally, structural knowledge of the targeted proteins (i.e. from crystallography studies) would clearly be advantageous when choosing where to integrate an internal tag sequence.

## SUPPLEMENTARY DATA

Supplementary Data are available at NAR Online.

SUPPLEMENTARY DATA
