# Supplementary material for: NLS-tagging: an alternative strategy to tag nuclear proteins
Source: Nucleic Acids Res. 2014 Sep 26;42(21):e163. doi: 10.1093/nar/gku869 (PMC4245968; doi:10.1093/nar/gku869)
Supplement: SUPPLEMENTARY DATA [file supp_42_21_e163__index.html]

NLS-tagging: an alternative strategy to tag nuclear proteins — NLS-tagging: an alternative strategy to tag nuclear proteins — SUPPLEMENTARY DATA 

# NLS-tagging: an alternative strategy to tag nuclear proteins

## SUPPLEMENTARY DATA

**Files in this Data Supplement:**

- SUPPLEMENTARY DATA
