# Supplementary material for: NLS-tagging: an alternative strategy to tag nuclear proteins
Source: Nucleic Acids Res. 2014 Sep 26;42(21):e163. doi: 10.1093/nar/gku869 (PMC4245968; doi:10.1093/nar/gku869)
Supplement: SUPPLEMENTARY DATA [file supp_gku869_nar-02091-met-g-2014-File008.pdf]

Supplementary Table 1: List of Fli-1 interacting proteins in MEL cells

Table depicting all the proteins interacting with Fli-1 in MEL cells and whose Mascott Score is at least 80 and two fold higher in 3xFlag-Bio(NLS)-Fli-1 than in MEL BirA cells.

Supplementary Table 2: Primers for plasmid construction

Primers used to amplify the different cDNA coding parts of 3xFlag-Bio(NLS)-Fli-1 and 3xFlag-Bio(NLS)-Irf2bp2 proteins

Supplementary Table 3: Primers used for RT-qPCR

Supplementary Table 4: Primers used for ChIP-qPCR

Supplementary Table 1 : List of Fli-1 interacting proteins in MEL cells

Protein	Unique Peptide 3xFlag-Bio(NLS)-Fli-1 cells	Unique Peptide MEL- BirA cells	Mascot score 3xFlag-Bio(NLS)-Fli-1 cells	Mascott score MEL-BirA cells
**Parp1**	31		1608	
**Etv6**	18		1008	
**C130039O16Rik**	14		841	
**Trrap**	19		814	
**Pfkl**	12	3	662	176
**Xrcc6**	11		596	
**Runx1**	10		547	
**Dek**	8	2	526	103
**Gata1**	7	4	464	210
**Vrk3**	8		439	
**Fermt3**	10	6	426	207
**Dido1**	8		415	
**Dnmt1**	7	3	352	147
**Pfkp**	6		343	
**Plk1**	8		342	
**Racgap1**	8		342	
**Dnttip1**	7		317	
**Snw1**	6	3	307	100
**Tal1**	6		301	
**Tada3**	5		261	
**Wiz**	6		252	
**Abcf2**	7		241	
**Mta2**	4	2	239	95
**Ldb1**	5		238	
**Nfix**	5		237	
**Ptbp1**	5	2	236	95
**Xrcc1**	4		213	
**Khdrbs1**	4	2	212	85
**Copb1**	5		203	
**Tfeb**	5		190	
**Ikzf1**	3		188	

Protein	Unique Peptide 3xFlag-Bio(NLS)-Fli-1 cells	Unique Peptide MEL- BirA cells	Mascot score 3xFlag-Bio(NLS)-Fli-1 cells	Mascott score MEL-BirA cells
Eif4a3	4		185	
Pfkm	4	2	185	70
Parp2	3		184	
Rbm17	4	1	178	52
Cpsf7	4	2	174	72
Znf710	3		166	
Vrk1	3		164	
Hdac2	3		159	
Taf6l	3		159	
Msh6	4		158	
Mta3	3		156	
Dnajc9	3		152	
Smarca4	3	1	152	47
Cbfb	3		151	
Taf5l	3		139	
Aplf	2		139	
Fxr1	3		131	
Rbm27	3		130	
Serbp1	2		127	
Bcor	2		126	
Tfe3	3		125	
Mtss1	3		123	
Banf1	2		123	
Hltf	2		123	
Baz1a	3		120	
Luc7l3	2		116	
Rcor1	3		115	
Hmmr	2		114	
Gfi1b	3		113	
Rbm25	3		112	
Cdk9	3		111	
Bcas2	3	1	109	45
Ints1	2		108	

Protein	Unique Peptide 3xFlag-Bio(NLS)-Fli-1 cells	Unique Peptide MEL- BirA cells	Mascot score 3xFlag-Bio(NLS)-Fli-1 cells	Mascott score MEL-BirA cells
**Xrn2**	3		107	
**Rbm33**	2		107	
**Ehmt2**	2		104	
**Fmr1**	2		102	
**Fxr2**	2		102	
**Rbm15**	3		101	
**Ubtf**	3		100	
**Tada1**	2		96	
**Prpf40a**	2		95	
**Psip1**	2		95	
**Smap2**	3		94	
**Tcf3**	3		94	
**Copg2**	2		94	
**Cbx3**	2		93	
**Pds5a**	2		93	
**Nfic**	2		91	
**Polb**	2		91	
**Ssrp1**	2		88	
**Cdk1**	2		87	
**Pogz**	2		86	
**Wdr82**	2		85	
**Zeb2**	2		85	
**Ubap2l**	2		84	
**Ssbp2**	1		84	
**Ssbp3**	1		84	
**Ssbp4**	1		84	
**Srsf10**	2		83	
**Srsf12**	2		83	
**Supt7l**	1		82	
**Ehmt1**	2		81	
**Sf1**	2		80	
**2810422J05Rik**	1		80	

Protein	Unique Peptide 3xFlag-Bio(NLS)-Fli-1 cells	Unique Peptide MEL- BirA cells	Mascot score 3xFlag-Bio(NLS)-Fli-1 cells	Mascott score MEL-BirA cells
**2810422J05Rik**	1		80	
**Taf10**	1		80	
**Uba52**	1		80	

Supplementary Table 2 : Primers for plasmid constructions

Primer name	Primer sequence
Sense 3xFlag (IRF2BP2)	5'-CGATATCGACTACAAGGATCACGACGAGACTACAAGGATC ACGACATCGACTACCAAGGACGATGACGATAAGCCTAGGA-3'
Antisense 3xFlag (IRF2BP2)	5'-GATCTCCTAGGCTTATCGTCATCGTCCTTGGTAGTCGATGT CGTGATCCTTGTAGTCTCGTCGTGATCCTTGTAGTCGATATCGG TAC-3'
Sense 3xFlag (Fli-1)	5'- GGCCGCGGTACCGACTACAAGGATCACGACGAGACTACAAGGA TCACGACATCGACTACCAAGGACGATGACGATAAGCTCGAGA-3'
Antisense 3xFlag (Fli-1)	5'GATCTCTCGAGCTTATCGTCATCGTCCTTGGTAGTCGATGTCG TGATCCTTGTAGTCTCGTCGTGATCCTTGTAGTCGGTACCGC-3'
Forward N-term Fli-1	5'GAGAGAGCGGCCGCATGGACGGGACTATTAAGGAGG-3'
Reverse N-term Fli-1	5'-GAGAGAGGTACCTGATCCACTCCTGCTGCG-3'
Forward C-term Fli-1	5'-GAGAGACTCGAGGGAGGAGGACTAAACGACATCTTCGAGG CTCAGAAGATCGAGTGGCATCAGCCAGTGAGAGTCAATGTCA-3'
Reverse C-term Fli-1	5'GAGAGAAGATCTCTCAAGGCACGTGGGTGT3'
Forward N-term IRF2BP2	5'-GAGAGAGGTACCATGGCCGCGGCGGTGGCGGT-3'
Reverse N-term IRF2BP2	5'-GAGAGAGATATCTTTAGACCCGTTGGCCCCGT-3'
Forward C-term IRF2BP2	5'- GAGAGACCTAGGGGAGGAGGACTAAACGACATCTTCGAGGCT CAGAAGATCGAGTGGCATGCAGTTGCAAGGACAGCAAG-3'
Reverse C-term IRF2BP2	5'-GAGAGAAGATCTTCACGAGTCTCTCTCCTTCT-3'

Supplementary Table 3 : Primers RT-qPCR

Primer name	Primer sequence
Forward β-globin	5’- CAGGCTGCTGGTTGTCTA-3’
Reverse β-globin	5’-GAGGCTGTCCAAGTGATTC-3’
Forward sfpi-1	5’-AGAGCTATACCAACGTCCAA-3’
Reverse sfpi-1	5’-GGAAGTTCTCAAACTCGTTG-3’
Forward Alas-2	See reference 2
Reverse Alas-2	See reference 2

Supplementary Table 4 : Primers for ChIP-qPCR

Primer name	Primer sequence
**Forward Nip7 promoter**	5’- TCACGGAGAATTAGGCTAGA-3’
**Reverse Nip7 promoter**	5’-GGTCTCTTCTTCAGTCAAGG-3’
**Forward Fli-1 promoter**	5’-CGATCGCAAAGTGAAGTC-3’
**Reverse Fli-1 promoter**	5’-CCTGTGCACGTTTGTTGT-3’
**Forward Tgfb1 locus**	5’-GGTGTCAGTAGCTTCTCCAG-3’
**Reverse Tgfb1 locus**	5’-GAAATGGGGTGACATAGAGA-3’
**Forward Myb -36 kb enhancer (BS1)**	Stadhouders et al., 2012
**Reverse Myb -36 kb enhancer (BS1)**	Stadhouders et al., 2012
**Forward Myb -68 kb enhancer (BS5)**	Stadhouders et al., 2012
**Reverse Myb -68 kb enhancer (BS5)**	Stadhouders et al., 2012
**Forward β-amylase locus**	5’-CTCCTTGTACGGGTTGGT-3’
**Reverse β-amylase locus**	5’-AATGATGTGCACAGCTGAA-3’
